# A Causal Treatment for X-Linked Hypohidrotic Ectodermal Dysplasia: Long-Term Results of Short-Term Perinatal Ectodysplasin A1 Replacement

**DOI:** 10.3390/ijms24087155

**Published:** 2023-04-12

**Authors:** Holm Schneider, Christine Schweikl, Florian Faschingbauer, Smail Hadj-Rabia, Pascal Schneider

**Affiliations:** 1 Center for Ectodermal Dysplasias & Department of Pediatrics, University Hospital Erlangen, Loschgestr. 15, 91054 Erlangen, Germany; 2 Department of Obstetrics and Gynecology, University Hospital Erlangen, Universitätsstraße 21-23, 91054 Erlangen, Germany; florian.faschingbauer@uk-erlangen.de; 3 Department of Dermatology and Reference Center for Rare Skin Diseases (MAGEC), Institut Imagine, Université de Paris-Centre, Hôpital Necker-Enfants Malades, 149 Rue de Sèvres, 75743 Paris, France; smail.hadj@inserm.fr; 4 Department of Immunobiology, University of Lausanne, Chemin des Boveresses 155, 1066 Epalinges, Switzerland; pascal.schneider@unil.ch

**Keywords:** ectodermal dysplasia, ectodysplasin A, AlphaLisa, protein replacement, sweat glands, tooth development, prenatal therapy

## Abstract

X-linked hypohidrotic ectodermal dysplasia (XLHED), caused by a genetic deficiency of ectodysplasin A1 (EDA1), is a rare developmental disorder of ectodermal derivatives such as hair, sweat glands, and teeth. The absence of sweat glands and perspiration can evoke life-threatening hyperthermia. As molecular genetic findings are not always conclusive, the concentrations of circulating EDA1 may help to distinguish between total and partial EDA1 deficiencies. We previously treated nine male patients with obvious signs of XLHED with a recombinant EDA1 replacement protein, Fc-EDA, either shortly after birth (*n* = 3) or by prenatal administration in gestational week 26 and beyond (*n* = 6). Here, we present the long-term follow-up for up to six years. In patients who had received Fc-EDA after birth, neither sweat glands nor sweating ability were detected at the age of 12–60 months. In contrast, prenatal EDA1 replacement resulted in ample sweat gland development and pilocarpine-inducible sweating in all treated subjects, who also attained more permanent teeth than their untreated affected relatives. Normal perspiration has persisted for six years in the two oldest boys treated repeatedly with Fc-EDA in utero. When they had a sauna, adequate thermoregulation was evidenced. Lower sweat production after single prenatal dosing may indicate a dose–response relationship. The absence of circulating EDA1 in five prenatally treated subjects proved that these children would have been unable to perspire if they had been left untreated. The sixth infant was shown to produce an EDA1 molecule that, albeit interacting with its cognate receptor, cannot activate EDA1 signaling. In conclusion, a causal treatment of XLHED before birth is feasible.

## 1. Introduction

Ectodermal dysplasias are congenital genetic conditions affecting the development and/or homeostasis of two or more derivatives from the embryonic ectoderm, such as hair, teeth, nails, and certain glands in the skin and mucous membranes [1,2]. So far, no curative treatment is available. X-linked hypohidrotic ectodermal dysplasia (XLHED), the most frequent of these conditions, comprises congenital anomalies, for example, the absence of sweat glands which may become life-threatening in the first year of life [3,4] but also beyond infancy. The inability to perspire poses affected individuals at risk of severe hyperthermia during heat or sun exposure, febrile illness, or intense physical activity.

XLHED is caused by the lack of ectodysplasin A1 (EDA1), a transmembrane protein of the tumor necrosis factor (TNF) family [5]. EDA1 activates a signaling pathway downstream of Wnt/β-catenin signaling that is mediated by nuclear factor kappa B (NF-κB). It has been shown to be essential for the proper differentiation of ectodermal placodes, the precursors of eccrine sweat glands, hair follicles, and other skin appendages, during prenatal development [6]. EDA1 deficiency leads to dental abnormalities or missing teeth and is further associated with impaired gland development in the eye (lacrimal and meibomian glands), the oral cavity, and in mucous membranes of the upper respiratory tract, giving rise to dry eye problems, hyposalivation, a dry, crusty nose, a hoarse voice, reduced mucociliary clearance, frequent airway infections, and issues such as allergic asthma [7,8].

In an attempt to establish a molecular therapy for XLHED, we generated and studied a recombinant EDA1 replacement protein which was shown to prevent XLHED in *Tabby* mice [9,10], a well-characterized disease model [11], and mitigated the disorder in affected dogs [12,13,14]. We evaluated this protein, Fc-EDA, in first multicenter clinical trials on human patients (ClinicalTrials.gov identifiers: NCT01564225 and NCT01775462) [15] and, based on requests for compassionate use, also outside clinical studies [15,16]. The latter was due to the fact that reliable prenatal diagnosis of XLHED became established [17,18] and Fc-EDA could be administered even before birth, during the natural time window for sweat gland development [19]. The recombinant protein was injected into the amniotic fluid which is swallowed by the fetus; it enters the fetal bloodstream after binding to the neonatal Fc receptor [16] already present in the fetal gut in preparation for the uptake of antibodies from mother’s milk. Between 2013 and 2021 nine male infants with XLHED were treated at the Center for Ectodermal Dysplasias in Erlangen, Germany, either postnatally or by intra-amniotic administration of Fc-EDA at 26 weeks of pregnancy and beyond. Promising results of the prenatal approach were published five years ago [16].

Here, we report the findings of a long-term follow-up study that strongly support further clinical investigation of the efficacy and safety of EDA1 replacement in utero (ClinicalTrials.gov identifier: NCT04980638) as a new means of treating a genetic disability.

## 2. Results

### 2.1. Induction of Sweat Gland Development and Sweating Ability

In our center, treatment with Fc-EDA was considered only for male individuals hemi-zygous for a null mutation in the X-chromosomal gene *EDA* (NM_001399.45), which is known to result in anhidrosis. Details on the genotypes of the nine patients and the timing of the intervention(s) are summarized in Table 1.

**Figure 1:**
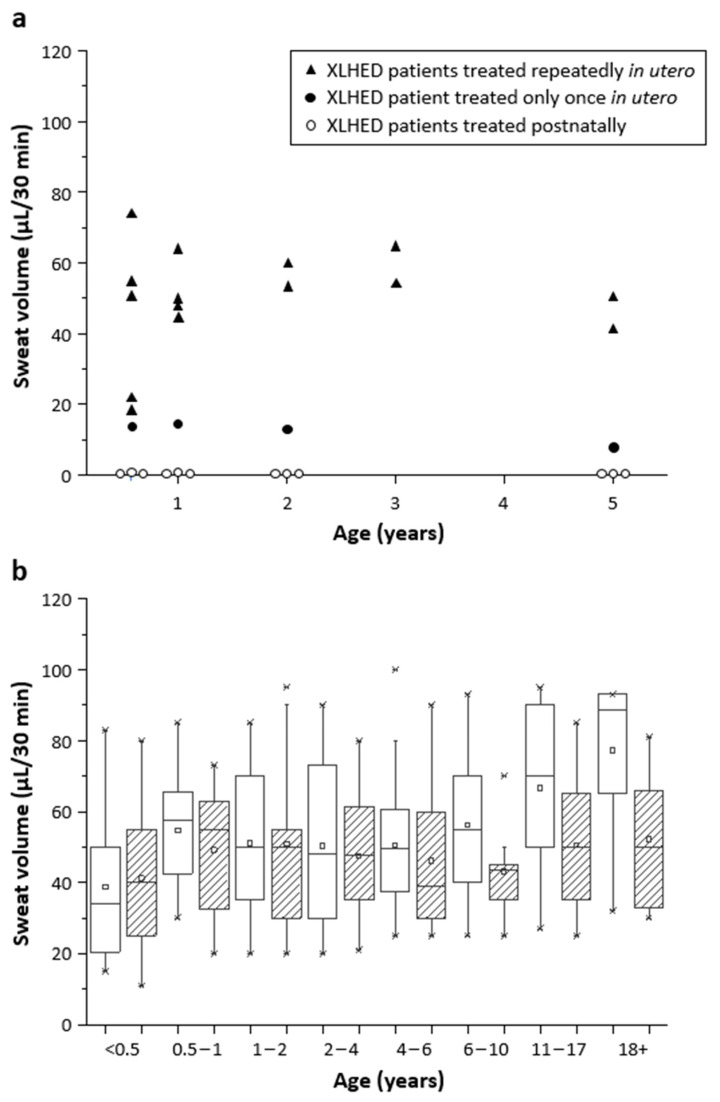
Chemically stimulated sweating. (**a**) Pilocarpine-induced sweat volumes were collected within 30 min in XLHED-affected infants who received prenatal treatment with Fc-EDA (black triangles and black circles) or postnatal injections of this replacement protein (white circles). (**b**) Pilocarpine-induced sweat production in male control subjects (white boxes; *n* = 174) and female subjects (striped boxes; *n* = 105) of different age groups. The edges of the box denote the 25th and 75th centiles. Crosses indicate the minimum and maximum volumes measured in the individual age group. ▫, mean; ─, median.

Untreated affected relatives (older brother, uncle, or grandfather) served as genotype-matched controls. Subjects N1, N2, and N3 who had received intravenous injections of Fc-EDA as neonates neither developed a significant number of sweat glands (as assessed by reflectance confocal laser-scanning microscopy of the soles) nor visible sweat secretion. Scheduled re-testing up to the age of five years confirmed the absence of pilocarpine-inducible sweat production (Figure 1a). In contrast, all subjects treated with at least two doses of Fc-EDA in utero had normal plantar sweat pore densities [15] (data of P4, P5, and P6 not shown), and those who were examined repeatedly presented with normal or near-normal chemically stimulated sweating (Figure 1), except for one measurement (7 µL/30 min) in subject P3 who had received only a single intra-amniotic injection.

**Figure 2:**
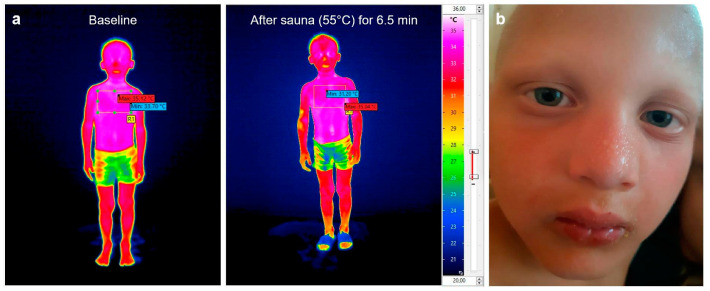
Documentation of thermal sweating. (**a**) Whole-body skin thermography of a 5-year-old prenatally treated XLHED patient (subject P1) before and after having a sauna. Efficient perspiration is indicated by skin temperatures lower than 32.5 °C (red to yellow color), for example at the nose, the right arm, both hands, and the lower legs. (**b**) Visible thermal sweating on the face of this patient when leaving the sauna: the nose and the forehead were particularly covered by beads of sweat.

The parents of all six subjects who had received Fc-EDA in utero reported that the skin of various body regions of their sons was repeatedly found to be moist, depending on the environmental temperature and physical activity. To confirm these parental observations of thermal sweating, we accompanied subjects P1 and P2 when they had a sauna together with their father and were able to document obvious perspiration by infrared thermography (Figure 2a) and photos of sweating body regions (Figure 2b shows an example). Body core temperatures of both treated boys remained below 38 °C.

Upon independent stimulation by exercising for 15 min, subjects P1, P2, and P3 produced relevant amounts of sweat on the back (21, 25, and 18 µL/cm^2^, respectively), comparable with those of healthy control infants, while their untreated older brothers did not sweat at all and have never be willing to set foot in a sauna.

Normal perspiration has been detectable for more than six years in the two oldest boys treated repeatedly with Fc-EDA in utero (58 and 67 µL/30 min at the last time-point investigated) and sufficient thermoregulation has enabled normal outdoor activities and high levels of fitness all year long. This is in sharp contrast to the absence of sweating and the poor thermoregulation in the untreated affected brother, as reported by the parents. Subject P3 (who received a single intra-amniotic injection of the EDA1 replacement protein) has also enjoyed numerous activities and physical challenges even on hot summer days, but the lower pilocarpine-inducible sweat production and his drier skin may indicate a dose–response relationship for the prenatal treatment.

### 2.2. Impact of EDA1 Replacement on Other XLHED Symptoms

Up to the last study visit at the age of 60 months, the three subjects who had received early postnatal treatment with Fc-EDA showed the classical signs of XLHED, including oligodontia, anhidrosis, and hyperthermic episodes during the summer; dry eyes; frequent nosebleeds and airway infections; a lack of saliva; hoarse voice; and dry skin (Table 2). Their condition did not differ from that of untreated XLHED patients of the same age [8].

In contrast, patients P1, P2, and P3 who underwent treatment with the replacement protein in utero have so far neither experienced hyperthermic episodes during the summer nor any significant eye, nose, throat, or respiratory issues (Table 2). 

However, the impact of prenatal Fc-EDA dosing on permanent teeth does not appear to be optimal yet, and there have been no corrective effects on the development of scalp hair and primary teeth which are both ectodermal structures that form during early embryogenesis.

**Figure 3:**
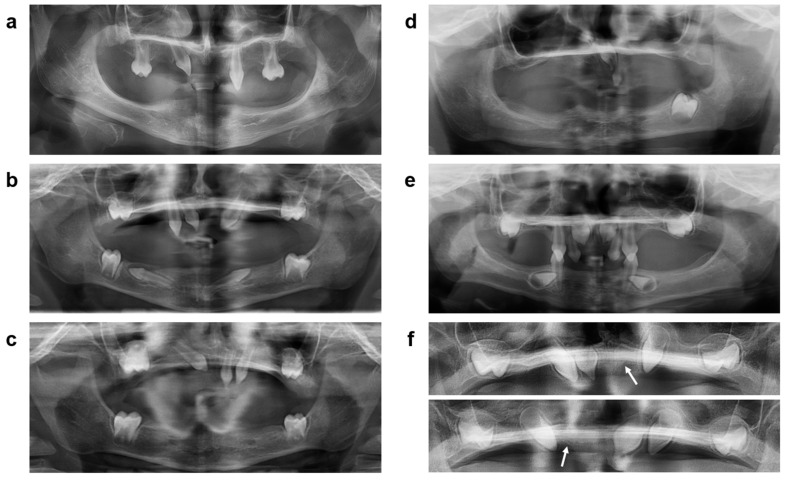
Panoramic dental radiographs of the untreated XLHED-affected brother (**a**) of subjects P1 and P2 compared with the radiographs of these Fc-EDA-treated subjects at preschool age (**b**,**c**) and of the untreated affected brother of P3 (**d**) compared with the younger sibling 6 years after his prenatal treatment with Fc-EDA (**e**). Two primary tooth germs ((**f**), arrowed) that had been detectable in P1 and P2 in early infancy did not undergo complete mineralization and did not erupt.

Dental radiographs of untreated affected siblings compared with those of subjects P1, P2, and P3 at preschool age (Figure 3a–e) revealed a total of 4 and 2 permanent teeth in the untreated boys, but 9, 7, and 8 tooth buds of the permanent dentition, respectively, in their treated brothers (in agreement with magnetic resonance imaging data from the first year of life). The primary tooth germs of P1 and P2 (one in each subject) had not developed any further (Figure 3f), so in both subjects no teeth erupted before the age of five years. Interestingly, buds of all four permanent first molars were detected in P1 and P2, suggesting a particular stimulation of the development of permanent molars by prenatal treatment with Fc-EDA. Subject P3, who had received only a single Fc-EDA dose, later grew four primary teeth; he lacked, however, two buds of permanent first molars (Figure 3e).

### 2.3. Absence of Functional EDA1 in the Circulation of Treated Patients

**Figure 4:**
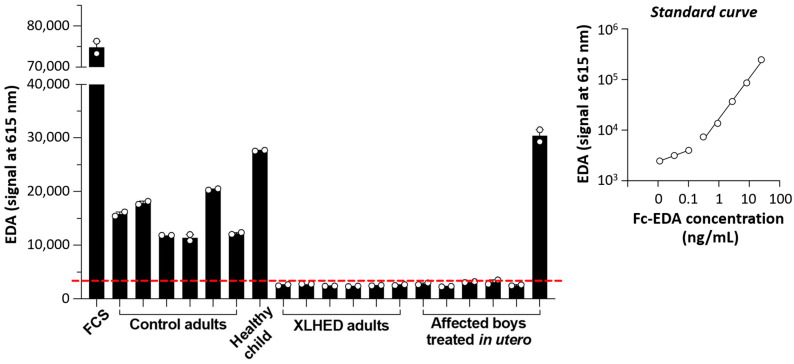
Levels of circulating EDA as determined by AlphaLisa. The measurements, always conducted in duplicates, were reproducible in three independent experiments. A representative assay is shown here. Signals below the dashed red line are considered as background. The standard curve was generated with Fc-EDA diluted in serum from an adult male subject with XLHED. FCS, fetal calf serum.

To confirm that the therapeutic effects were indeed mediated by the replacement protein Fc-EDA, we investigated the concentrations of circulating ectodysplasin A (EDA; referring to both isoforms, EDA1 and EDA2) in serum samples of subjects P1–P6 taken after birth. Fetal calf serum (FCS) and specimens from healthy individuals including one child served as positive controls. Sera from adults with the full-blown phenotype of XLHED were analyzed in parallel (negative control). The results are shown in Figure 4.

**Figure 5:**
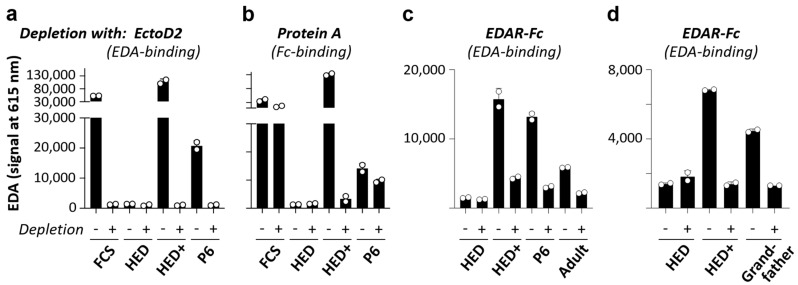
Circulating EDA, as determined by AlphaLisa, following pre-depletion with EctoD2, protein A, or EDAR-Fc. (**a**) Fetal calf serum (FCS), serum from an adult subject with XLHED, either pure (HED) or spiked with Fc-EDA (50 ng/mL; HED+), and serum from subject P6 were depleted twice with EctoD2 (anti-EDA)-coupled beads (+) or TACI-Fc-coupled beads (-). EDA in flow-through fractions was measured by AlphaLisa. (**b**) Same as (**a**) except that serum was depleted four times with Protein A-coupled beads (+) or Sepharose 6B beads (-). The beads were then acid-eluted and released immunoglobulins were quantified. No immunoglobulin was detected in the 4th elution of Protein A, indicating successful target depletion. There was no retention of immunoglobulins on Sepharose 6B. (**c**,**d**) Same as (**a**) except that sera were depleted once with EDAR-Fc (+) or with TACI-Fc (-) and that serum from an adult subject with XLHED was spiked with Fc-EDA at a concentration of 5 ng/mL (HED+). Measurements were conducted in duplicates. Adult, healthy adult control.

The absence of circulating EDA in five subjects treated in utero (P1–P5) proved that these boys carry *EDA* null mutations and would be unable to sweat if they had been left untreated. Surprisingly, a signal corresponding to a large amount of EDA, similar to that obtained for the healthy child, was measured in the serum of 3-month-old subject P6. This signal could be depleted with the antibody EctoD2 (binding to EDA) but not by depleting molecules with Fc polypeptide chains, such as immunoglobulins or Fc-EDA. Hence, the EDA detected in the serum of infant P6 behaved similarly to endogenous EDA (tested with FCS) and not like Fc-EDA (tested with serum from an adult XLHED patient that had been spiked with Fc-EDA) (Figure 5a,b). This suggested that, in contrast to the prediction of a truncated non-functional EDA1 protein that would lack the domain detected in our assay (see Table 1), subject P6 was able to produce a normal quantity of endogenous EDA. The experimental results also allowed the conclusion that the unexpected EDA signal cannot be explained by long-term remnants of Fc-EDA in the subject’s circulation.

**Figure 6:**
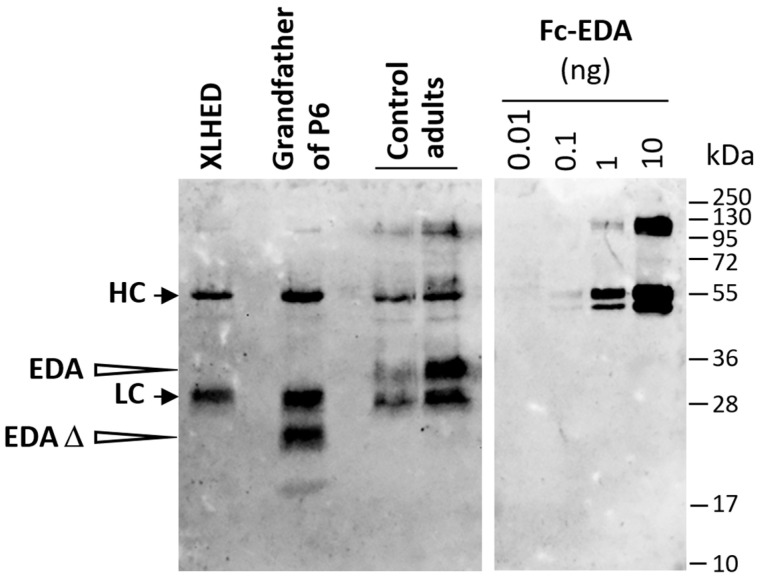
Expression of a shortened EDA molecule in an affected relative of P6. Sera from a pool of adult subjects with XLHED who all carry *EDA* null mutations (no EDA1 expression), from the grandfather of P6 and also from two healthy adults were investigated by immunoprecipitation with a mix of two anti-EDA monoclonal antibodies, EctoD2 and EctoD3, that both recognize the native C-terminal domain of EDA. Subsequent Western blotting with the antibody Renzo-2 recognizing the denatured C-terminal domain of EDA was performed. Increasing amounts of recombinant Fc-EDA were loaded onto the same gel as positive controls. The positions of molecular weight markers (in kDa) are indicated. HC and LC, heavy and light chains of the antibodies used for immunoprecipitation; EDA, processed soluble form of wild-type EDA; EDA Δ, shorter form of EDA in serum from the grandfather of P6. This experiment was conducted twice with similar results.

Next, we conducted a pre-depletion experiment using EDAR-Fc to assess the receptor-binding capability of the EDA protein produced by subject P6. In this assay, the signal could be depleted with EDAR-Fc but not with a control protein, the transmembrane activator, calcium-modulator and cyclophilin ligand interactor (TACI) fused to Fc (Figure 5c). The experiment was then repeated with serum from the XLHED-affected grandfather of subject P6 who shows severe symptoms of ectodermal dysplasia including anhidrosis. The grandfather’s serum also contained EDA capable of binding to EDAR-Fc (Figure 5d). Thus, affected male individuals in the family of subject P6 seemed to produce an EDA1 molecule that—although interacting with its receptor—cannot activate EDA1 signaling and, therefore, does not prevent the full-blown phenotype of XLHED. As this might be explained by alternative mRNA splicing to skip exon 4 (180 bp, encoding the entire collagen domain), we performed an immunoprecipitation followed by Western blotting, which proved that the EDA protein in the grandfather’s serum is really shorter than that of healthy control subjects (Figure 6). The same experiment with material from P6 could not be conducted as we did not have enough serum from the infant.

## 3. Discussion

Approximately 20% of patients with XLHED are able to sweat and regulate their body core temperature to some degree [20,21], which can be explained by *EDA* mutations allowing a residual activity of EDA1 (hypomorphic mutations). However, none of the nine subjects investigated in this study carried a hypomorphic mutation. They are all hemizygous for a null mutation in *EDA* known to cause anhidrosis. In the case of subject P6, where detectability of EDA protein in the serum raised some doubts, both anhidrosis and circulating EDA were evident in another male family member with the same *EDA* variant, suggesting functional impairment of the protein. The familial *EDA* variant, c.562_589del in exon 4, induces a frameshift and should result in the absence of EDA1 protein, while our data demonstrate the presence of an EDA molecule capable of binding to EDAR in two family members. As the grandfather shows the full-blown phenotype of XLHED including anhidrosis, this EDA molecule is presumably inactive, despite binding to its cognate receptor. We hypothesized that the mutation induces alternative RNA splicing to skip exon 4. This would preserve the furin cleavage site as well as the TNF homology domain which is responsible for the formation of homotrimers and binding to EDAR, but would result in an EDA protein lacking the entire collagen domain and, therefore, being unable to activate the signaling pathway in vivo [22,23]. Immunoblots demonstrated that the EDA molecule in the grandfather’s blood is really shortened, thus confirming our hypothesis.

Anhidrosis and heat intolerance, the most severe disability related to XLHED [3,4,8], appear to be amenable to timely EDA1 replacement therapy. The long-term results presented here confirm the previously reported failure of early postnatal treatment with Fc-EDA in human patients but also the efficacy of prenatal intra-amniotic delivery of this recombinant protein [16]. Such treatment in utero normalized thermoregulation in affected infants, protecting them from potentially life-threatening hyperthermia and febrile seizures [8] that have the potential to cause irreversible brain damage. In none of the six cases has XLHED-related hospitalization been required thus far. If persisting further, the therapeutic effect will have a major impact on the children’s ability to attend school and participate in physical activities throughout the year [24].

To our knowledge, there is no reason to assume that once sweat glands are normally formed they would not work permanently. Although sweating varies across the skin surface [25], we were surprised that pilocarpine-induced sweat production on the forearm of subject P3 at the age of five years was only half of that measured at the age of six months (7 vs. 14 µL), while infrared thermography after exercising for 15 min showed efficient perspiration on his legs, trunk, and head. As this boy’s reported heat tolerance has remained unchanged over five years, the difference in sweat volumes might be explainable by an irregular distribution and function of sweat glands. The higher variability in perspiration may be due to the single dosing, as all subjects treated with two and more intra-amniotic injections of the replacement protein did not show a decrease in pilocarpine-induced sweating over time.

The three more recently treated patients, P4–P6, who produced pilocarpine-induced sweat volumes within the normal range, comparable to P1 and P2, will also be followed until school age.

Proper assessment of the impact of repeated Fc-EDA administration in utero on tooth development requires a longer follow-up that also covers permanent dentition. The missing eruption of primary teeth despite radiographic detectability of at least one primary tooth germ in subjects P1 and P2, respectively, may indicate an inhibition of the primary dentition by prenatal treatment with Fc-EDA, which might have been less relevant in subject P3 because of single prenatal dosing. Considering the small number of treated patients who are old enough for thorough examination of tooth development, it seems premature to draw conclusions at this stage. As permanent teeth provide more long-term benefit than primary teeth anyway, we will not refrain from treating patients with up to three doses of Fc-EDA in the current EDELIFE clinical trial [26]. 

The significance of all our findings in the small series of individual patients reported here remains to be determined in the ongoing EDELIFE trial on a larger group of boys with XLHED who are to receive protein replacement therapy in utero.

## 4. Materials and Methods

### 4.1. Investigational Medicinal Product

Fc-EDA, a recombinant fusion protein consisting of the receptor-binding portion of EDA1 and the Fc domain of human immunoglobulin G1, was produced according to Good Manufacturing Practice regulations and provided as a frozen, sterile drug product with concentrations of 5 or 10 mg/mL in 20 mM sodium phosphate, 300 mM sodium chloride, pH 7.2, and 0.02% Polysorbate 20 (*w*/*v*) either by Edimer Pharmaceuticals, Cambridge, MA, USA (for all patients treated until 2016), or by the EspeRare Foundation, Geneva, Switzerland (for patients treated after 2016).

### 4.2. Therapeutic Interventions Studied

Subjects N1, N2, and N3 were treated within a multicenter phase 2 dose-escalation study to evaluate the safety, pharmacokinetics, immunogenicity, and pharmacodyna-mics/efficacy of Fc-EDA administered to newborn infants with XLHED (ClinicalTrials.gov Identifier: NCT01775462). Each subject received 5 equal doses of undiluted study drug administered intravenously over 14 days by slow infusion with a syringe pump system into a peripheral or central vein via an intravenous catheter on days 0, 4, 7, 11, and 14 (window of ±24 h acceptable with a minimum of two days between any two doses; infusion rate not to exceed 5 mL/kg per hour). Subject N1 received 3 mg/kg. The other two subjects were dosed with 10 mg/kg, respectively, in total volumes between 2.1 and 5.6 mL. Further details and the first results of this clinical trial were reported previously [15]. The interventional study and a subsequent follow-up (extension) study were approved by the Ethics Committee of the University Erlangen-Nürnberg (codes 76_13 Az and 20_14 Az, respectively) and conducted according to Good Clinical Practice guidelines.

In six cases of male fetuses, each with a family history of XLHED and the prenatal diagnosis of this disorder (one pair of twins and four single fetuses), the parents requested compassionate use of Fc-EDA in utero which was approved by the clinical ethics committee of the University Hospital Erlangen on a case-by-case basis, between February 2016 and January 2021. Fc-EDA (100 mg/kg of estimated fetal weight; volumes of 14–28 mL) was injected under ultrasound guidance into the amniotic cavity of each fetus. One to three doses were administered, the first dose at week 26 of pregnancy. Details of the procedure and early data indicating the success of this treatment in the first three infants (P1, P2, and P3) have already been reported by our group [15]. Subjects P4, P5, and P6 received three doses of Fc-EDA, respectively, with intervals of two to four weeks between doses.

The long-term follow-up of the treated subjects was approved by the Ethics Committee of the University Erlangen-Nürnberg (code 95_14 B) on November 30, 2016. Written informed consent of both parents to the intervention and the participation of their child in the long-term study was obtained for each subject.

### 4.3. Assessment of Sweat Gland Density and Function

Conventional dermatoscopy using a magnifying glass and reflectance confocal microscopy with a VivaScope 1500 (Caliber Imaging and Diagnostics, Inc., Andover, MA, USA) was used to determine the sweat gland density at the soles of treated and control subjects [27]. Sweat duct counts in an area of 4 mm^2^ were extrapolated to the whole body surface area according to the Mosteller formula [28]. Chemically induced sweating on the forearms (in an area of 57 mm^2^) was stimulated by pilocarpine iontophoresis using the Macroduct Sweat Collection System 3700 (ELITech Group, Puteaux, France) and quantified by volumetry with micropipettes [8,15], a method validated in our laboratory. At the age of three to five years the subjects also participated in age-appropriate indoor exercising at a room temperature of 24.4 °C for 15 min, during which sweat collection from an area of 115 cm^2^ on the subject’s back into commercially available sweat pads (MyDry, PHC Premium Hygiene and Cosmetics GmbH, Bielefeld, Germany) took place, followed by gravimetric quantitation of the fluid volume in the pad with an analytical laboratory balance (Sartorius, Göttingen, Germany).

### 4.4. Infrared Thermography

Non-contact thermal imaging was performed using the VarioCAM HDx 625 infrared camera (InfraTec GmbH, Dresden, Germany). Infrared pictures of the subjects (full body images) were taken before and after a 6.5-min sauna session at 55 °C. This allowed the visualization of parts of the skin that cooled down by the evaporation of sweat (color change to red and orange). The body core temperature was measured in parallel with an infrared ear thermometer (ThermoScan 7, Braun Healthcare, Lausanne, Switzerland).

### 4.5. Determination of EDA Concentrations in the Blood

The EDA concentration in 5 µL of serum was quantified in duplicate by AlphaLisa, as described previously [29]. In brief, samples were mixed with biotinylated EctoD3 [28] and with EctoD2 (mouse immunoglobulin G1 recognizing EDA) [30] acceptor beads and incubated for one hour in the dark. Streptavidin donor beads were added and the signal was recorded 10 min later using an Enspire plate reader (Perkin-Elmer, Schwerzenbach, Switzerland). The final assay volume was 50 µL with biotinylated EctoD3 at 0.3 mM, EctoD2 beads at 5 µg/mL, and streptavidin beads at 25 µg/mL. Standard curves were generated with Fc-EDA diluted in serum from an adult male subject with the full-blown phenotype of XLHED.

Pre-depletion experiments were conducted in which serum (50 µL) was added to pelleted Protein A-Sepharose (GE Healthcare, Munich, Germany) or control Sepharose 6B beads (Sigma-Aldrich, Taufkirchen, Germany) (5 µL), mixed, and then left for 30 min at room temperature. Samples were loaded into homemade minicolumns [31] and the flow-through was collected. The beads were washed with phosphate-buffered saline (PBS) and eluted with 100 µL of citrate-NaOH (50 mM, pH 2.7). Immunoglobulins in the eluate were quantified by absorbance measurement at 280 nm. The flow-through was depleted three more times on Protein A-Sepharose or Sepharose 6B beads. Pre-depletion experiments with EctoD2, TACI-Fc or EDAR-Fc: EctoD2, and EDAR-Fc (containing amino acids 1–183 of human EDAR fused to the Fc fragment of human IgG1) or TACI-Fc (containing amino acids 31–110 of human TACI fused to the Fc fragment of hIgG1) were coupled at 2 mg/mL to NHS-Sepharose (GE Healthcare) according to the manufacturer’s instructions. The depletion of 50-µL serum samples was performed as described for Protein A, except that they were conducted twice for one hour with EctoD2 (and TACI-Fc control) and once for one hour with EDAR-Fc (and TACI-Fc control).

### 4.6. Immunoprecipitation of Circulating EDA and Western Blot

EDA was immunoprecipitated from 1 mL of human serum for 16 h at 4 °C using 7.5 µL of NHS-Sepharose beads (GE Healthcare) covalently coupled to a mix of EctoD2 and EctoD3 (2 mg/mL). Beads were collected in mini-columns, washed 3× with 100 µL of PBS containing bovine serum albumin (BSA; 10 µg/mL), eluted with 15 µL of citrate-NaOH (50 mM, pH 2.7, containing BSA at 10 µg/mL), and neutralized with 5 µL of 1 M Tris-HCl containing BSA (10 µg/mL). Western blotting under reducing conditions (30 mM dithiothreitol) was performed according to standard protocols, except that samples were heated for 3 min at 70 °C instead of 95 °C. The blot was revealed using the anti-EDA monoclonal antibody Renzo-2 at 1 µg/mL (produced in-house, also available from Enzo Life Sciences (Lausen, Switzerland; ALX-804-839), followed by application of a secondary horseradish peroxidase-coupled goat anti-mouse antibody diluted 1:8000 (Jackson Immunoresearch; 115-035-146) with subsequent incubation of the membranes with SuperSignal West Atto chemiluminescence reagent (Thermo Fisher Scientific, Ecublens, Switzerland; A38554) according to the manufacturer’s instructions, and imaging with an iBright CL1500 imaging system (Thermo Fisher Scientific).

## Figures and Tables

**Table 1:** Molecular and clinical characteristics of the study subjects.

Subject Code	Pathogenic *EDA* Variant	Predicted Molecular Effects	Functional Impact of the Mutation	Subject’s Age at First Treatment ^1^	Year of Treatment
N1	c.397-5858_502+3441dup; p.Gly168AspfsX10	Truncated, non-functional EDA1 protein	Anhidrosis	3 days	2013
N2	c.925-3C>G	Altered mRNA splicing	Anhidrosis	9 days	2014
N3	c.463C>T; p.Arg155Cys	Impaired furin cleavage	Anhidrosis	5 days	2015
P1	c.911A>G; p.Tyr304Cys	Insoluble EDA1 protein	Anhidrosis	GA 25 weeks + 4 days	2016
P2	c.911A>G; p.Tyr304Cys	Insoluble EDA1 protein	Anhidrosis	GA 25 weeks + 4 days	2016
P3 *	c.924+1dupG; p.V309GfsX8	Truncated, non-functional EDA1 protein	Anhidrosis	GA 26 weeks + 6 days	2016
P4	Deletion of exon 1	Nonsense-mediated decay	Anhidrosis	GA 25 weeks + 1 day	2020
P5	c.1133C>T; p.Thr378Met	Impaired receptor binding	Anhidrosis	GA 25 weeks + 6 days	2020
P6	c.562_589del; p.Pro188ArgfsX83	Truncated, non-functional EDA1 protein	Anhidrosis	GA 25 weeks + 2 days	2021

^1^ GA, gestational age; * received only a single intra-amniotic injection of Fc-EDA.

**Table 2:** The long-term impact of EDA1 replacement on XLHED symptoms, investigated at the age of 60 ± 3 months.

Subject	Body Length (cm) ^1^	Body Weight (kg) ^1^	Primary Teeth (Number)	Permanent Teeth (Number)	Pilocarpine- Induced Sweating (µL/30 min)	Episodes of Hyperthermia during the Summer?	Dry Eye Issues?	Nosebleeds (Number per Year)	Repeated Airway Infections?	Dry Mouth?	Hoarse Voice?	Dry Skin?
N1	106.5 14th perc.	18.7 42nd perc.	2	4	0	yes	yes	5	yes	yes	yes	yes
N2	109 26th perc.	17.6 24th perc.	8	5	0	yes	yes	50	yes	yes	yes	yes
N3	110 40th perc.	18.7 44th perc.	1	2	0	yes	yes	4	yes	yes	yes	yes
P3 (single dose)	108 41st perc.	18.8 56th perc.	4	8	7	no	no	2	no	no	no	yes
P1 (two doses)	105 10th perc.	16.2 11th perc.	1	9	41	no	no	0	no	no	no	no
P2 (two doses)	106 14th perc.	16.5 14th perc.	1	7	50	no	no	0	no	no	no	no

^1^ Length and weight percentiles (perc.) indicated.

## Data Availability

The data and statistical analyses supporting the reported results are either presented in this manuscript or available on request from the corresponding author.

## References

[1] Wright J.T., Fete M., Schneider H., Zinser M., Koster M.I., Clarke A.J., Hadj-Rabia S., Tadini G., Pagnan N., Visinoni A.F. (2019). Ectodermal dysplasias: Classification and organization by phenotype, genotype and molecular pathway. Am. J. Med. Genet. Part A.

[2] Peschel N., Wright J.T., Koster M.I., Clarke A.J., Tadini G., Fete M., Hadj-Rabia S., Sybert V.P., Norderyd J., Maier-Wohlfart S. (2022). Molecular pathway-based classification of ectodermal dysplasias: First five-yearly update. Genes.

[3] Freire-Maia N., Pinheiro M. (1990). Precocious mortality in Christ-Siemens-Touraine syndrome. Am. J. Med. Genet..

[4] Blüschke G., Nüsken K.-D., Schneider H. (2010). Prevalence and prevention of severe complications of hypohidrotic ectodermal dysplasia in infancy. Early Hum. Dev..

[5] Kere J., Srivastava A.K., Montonen O., Zonana J., Thomas N., Ferguson B., Munoz F., Morgan D., Clarke A., Baybayan P. (1996). X-linked anhidrotic (hypohidrotic) ectodermal dysplasia is caused by mutation in a novel transmembrane protein. Nat. Genet..

[6] Mikkola M.L., Thesleff I. (2003). Ectodysplasin signaling in development. Cytokine Growth Factor Rev..

[7] Dietz J., Kaercher T., Schneider A.T., Zimmermann T., Huttner K., Johnson R., Schneider H. (2013). Early respiratory and ocular involvement in X-linked hypohidrotic ectodermal dysplasia. Eur. J. Pediatr..

[8] Wohlfart S., Meiller R., Hammersen J., Park J., Menzel-Severing J., Melichar V., Huttner K., Johnson R., Porte F., Schneider H. (2020). Natural history of X-linked hypohidrotic ectodermal dysplasia: A 5-year follow-up study. Orphanet J. Rare Dis..

[9] Gaide O., Schneider P. (2003). Permanent correction of an inherited ectodermal dysplasia with recombinant EDA. Nat. Med..

[10] Hermes K., Schneider P., Krieg P., Dang A., Huttner K., Schneider H. (2014). Prenatal therapy in developmental disorders: Drug targeting via intra-amniotic injection to treat X-linked hypohidrotic ectodermal dysplasia. J. Invest. Dermatol..

[11] Blecher S.R. (1986). Anhidrosis and absence of sweat glands in mice hemizygous for the Tabby gene: Supportive evidence for the hypothesis of homology between Tabby and human anhidrotic (hypohidrotic) ectodermal dysplasia (Christ-Siemens-Touraine syndrome). J. Invest. Dermatol..

[12] Casal M.L., Lewis J.R., Mauldin E.A., Tardivel A., Ingold K., Favre M., Paradies F., Demotz S., Gaide O., Schneider P. (2007). Significant correction of disease after postnatal administration of recombinant ectodysplasin A in canine X-linked ectodermal dysplasia. Am. J. Hum. Genet..

[13] Mauldin E.A., Gaide O., Schneider P., Casal M.L. (2009). Neonatal treatment with recombinant ectodysplasin prevents respiratory disease in dogs with X-linked ectodermal dysplasia. Am. J. Med. Genet. Part A.

[14] Margolis C.A., Schneider P., Huttner K., Kirby N., Houser T.P., Wildman L., Grove G., Schneider H., Casal M.L. (2019). Prenatal treatment of X-linked hypohidrotic ectodermal dysplasia using recombinant ectodysplasin in a canine model. J. Pharmacol. Exp. Ther..

[15] Körber I., Klein O., Morhart P., Faschingbauer F., Grange D., Clarke A., Bodemer C., Maynard J., Maitz S., Huttner K. (2020). Safety and immunogenicity of Fc-EDA, a recombinant ectodysplasin A1 replacement protein, in human subjects. Br. J. Clin. Pharmacol..

[16] Schneider H., Faschingbauer F., Schuepbach-Mallepell S., Körber I., Wohlfart S., Dick A., Wahlbuhl M., Kowalczyk-Quintas C., Vigolo M., Kirby N. (2018). Prenatal correction of X-linked hypohidrotic ectodermal dysplasia. N. Engl. J. Med..

[17] Wünsche S., Jüngert J., Faschingbauer F., Mommsen H., Goecke T., Schwanitz K., Stepan H., Schneider H. (2015). Non-invasive prenatal diagnosis of hypohidrotic ectodermal dysplasia by tooth germ sonography. Ultraschall Med..

[18] Hammersen J., Wohlfart S., Goecke T.W., Köninger A., Stepan H., Gallinat R., Morris S., Bücher K., Clarke A., Wünsche S. (2019). Reliability of prenatal detection of X-linked hypohidrotic ectodermal dysplasia by tooth germ sonography. Prenat. Diagn..

[19] Ersch J., Stallmach T. (1999). Assessing gestational age from histology of fetal skin: An autopsy study of 379 fetuses. Obstet. Gynecol..

[20] Schneider H., Hammersen J., Preisler-Adams S., Huttner K., Rascher W., Bohring A. (2011). Sweating ability and genotype in individuals with X-linked hypohidrotic ectodermal dysplasia. J. Med. Genet..

[21] Massey H., House J., Tipton M. (2019). Thermoregulation in ectodermal dysplasia: A case series. Int. J. Environ. Res. Public Health.

[22] Schneider P., Street S.L., Gaide O., Hertig S., Tardivel A., Tschopp J., Runkel L., Alevizopoulos K., Ferguson B.M., Zonana J. (2001). Mutations leading to X-linked hypohidrotic ectodermal dysplasia affect three major functional domains in the tumor necrosis factor family member ectodysplasin-A. J. Biol. Chem..

[23] Swee L.K., Ingold-Salamin K., Tardivel A., Willen L., Gaide O., Favre M., Demotz S., Mikkola M., Schneider P. (2009). Biological activity of ectodysplasin a is conditioned by its collagen and heparan sulfate proteoglycan-binding domains. J. Biol. Chem..

[24] Hammersen J., Neukam V., Nüsken K.-D., Schneider H. (2011). Systematic evaluation of exertional hyperthermia in children with hypohidrotic ectodermal dysplasia: An observational study. Pediatr. Res..

[25] Machado-Moreira C.A., Smith F.M., van den Heuvel A.M., Mekjavic I.B., Taylor N.A. (2008). Sweat secretion from the torso during passively-induced and exercise-related hyperthermia. Eur. J. Appl. Physiol..

[26] Schneider H., Hadj-Rabia S., Faschingbauer F., Bodemer C., Grange D.K., Norton M., Cavalli R., Tadini G., Stepan H., Clarke A. (2023). Protocol for the phase 2 EDELIFE trial investigating the efficacy and safety of intra-amniotic ER004 administration to male subjects with X-linked hypohidrotic ectodermal dysplasia. Genes.

[27] Burger K., Schneider A.T., Wohlfart S., Kiesewetter F., Huttner K., Johnson R., Schneider H. (2014). Genotype-phenotype correlation in boys with X-linked hypohidrotic ectodermal dysplasia. Am. J. Med. Genet. Part A.

[28] Mosteller R.D. (1987). Simplified calculation of body-surface area. N. Engl. J. Med..

[29] Podzus J., Kowalczyk-Quintas C., Schuepbach-Mallepell S., Willen L., Staehlin G., Vigolo M., Tardivel A., Headon D., Kirby N., Mikkola M. (2017). Ectodysplasin A in biological fluids and diagnosis of ectodermal dysplasia. J. Dent. Res..

[30] Kowalczyk-Quintas C., Willen L., Dang A., Sarrasin H., Tardivel A., Hermes K., Schneider H., Gaide O., Donzé O., Kirby N. (2014). Generation and characterization of function-blocking anti-ectodysplasin A (EDA) monoclonal antibodies that induce ectodermal dysplasia. J. Biol. Chem..

[31] Schneider P., Willen L., Smulski C.R. (2014). Tools and techniques to study ligand-receptor interactions and receptor activation by TNF superfamily members. Meth. Enzymol..

